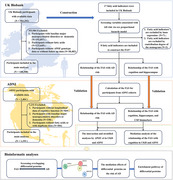# The blood lipidome fatty acid profile predicts the disease risk and clinical phenotypes of Alzheimer’s disease: associations from two prospective cohort studies

**DOI:** 10.1002/alz70861_108161

**Published:** 2025-12-23

**Authors:** Wei Xu, Liangyu Huang, Chen‐Chen Tan, Lan Tan

**Affiliations:** ^1^ Qingdao Municipal Hospital, Qingdao University, Qingdao, Shandong China; ^2^ Qingdao Municipal Hospital, Qingdao, 266071 China; ^3^ Qingdao Municipal hospital, Qingdao university, Qingdao, Shandong China

## Abstract

**Background:**

The association between fatty acids and the risk of developing Alzheimer's Disease (AD) has been a topic of growing interest but remains insufficiently understood. This study aims to construct and validate a comprehensive risk score derived from blood fatty acid indicators associated with AD risk and to uncover potential mechanisms.

**Methods:**

Data were obtained from the UK Biobank (UKB) (n = 148,308, mean age = 55.96, mean follow‐up: 12.3 years) and the Alzheimer's Disease Neuroimaging Initiative (ADNI) (n = 1,193, mean age = 73.50, mean follow‐up: 4.2 years). Lasso regression was used to construct a fatty acid score (FAS) associated with AD risk using UKB data. Cox proportional hazards regression and linear regression were employed to explore the relationships of FAS with AD risk, cognition, hippocampal volume, and/or cerebrospinal fluid markers. The interaction terms of FAS by *APOE* ε4 status were added in all analyses to test potential stratified effects. The causal mediation analyses, proteomic analyses, and bioinformatic analyses were conducted to elucidate the potential mechanisms.

**Results:**

Eight fatty acid components were included to construct the FAS. Higher FAS was associated with increased risk of AD in both UKB (HR = 1.298, *p* < 0.001) and ADNI (HR = 1.413, *p* = 0.006). Individuals with higher FAS tended to have lower hippocampal volume (P < 0.001) in UKB and to exhibit faster hippocampal atrophy (P = 0.002) and cognitive decline (MEM, *p* < 0.001) in ADNI. These associations were more pronounced in *APOE* ε4 carriers. Hippocampal volume partly mediated the association between FAS and cognitive decline (proportion ranging from 19.2% to 38.6%). The cytokines and inflammatory response, glial cell‐derived neurotrophic factor receptor signaling pathway, and pathways related to nervous system development could be potential mechanisms underpinning the association of FAS with AD.

**Conclusion:**

The blood fatty acid could help predict AD, though the causal relationship warrants further validation.